# Some Cases Curious of Diagnosis

**Published:** 1880-10-20

**Authors:** A. F. Kinne

**Affiliations:** Michigan


### Southern Medical Record.

EDITORS:

T. S. POWELL, M.D. W. T. GOLDSMITH, M.D. R. C. WORD, M.D.

*R. C. WORD, M.D., Managing Editor.*

☞All Communications and Letters on Business connected with the RECORD must be addressed to the Managing Editor.

VOL. X. ATLANTA, GA., OCTOBER 20, 1880. NO. 10.

### ORIGINAL AND SELECTED ARTICLES.

### SOME CASES CURIOUS OF DIAGNOSIS.

BY A. F. KINNE, A.M., M.D., OF MICHIGAN.

*Case* 1.—I was requested some years since, by my friend, Dr. N. Webb, of this city, to see a child of his, a well grown and healthy girl, aged 12 years, and help him, if I could, in making a diagnosis. At the time of my arrival, at 10 o'clock of a Saturday night, she had had a fever, all the time increasing rather than remitting, for fifty-three hours. What kind of a fever was it?

To give me all the facts that could have any possible bearing upon the case, the Doctor stated that on the previous Monday, the child had got scalded in such a way as to blister the posterior aspect of one of the lower extremities, from above the middle of the thigh down to the ankle. But the burn did not prove to be deep, and had given him no trouble. He had taken especial care that the loosed cuticle should not be disturbed, and in two or three days, with the use of only ordinary remedies, a new one had formed beneath it, and the soreness was gone.

Between the burn, therefore, and the fever, which came on with a distinct chill, at about 5 o'clock Thursday afternoon, he apprehended no connection—except perhaps that so extensive a lesion might have had some tendency to reduce the tone of the system. He took it to be an ordinary bilious attack and gave a mild cathartic, intending to follow it up with quinine as soon as the expected remission began to show itself. But the fever did not abate. On the other hand, it was higher the next afternoon than it was in the beginning. But as there were no noticeable symptoms of any local disease, he understood this increase of fever simply to mean that the case was a hard one, and added fever powders to the treatment, and waited, hoping to see some signs of a much-wished-for remission further on.

But in this expectation he was doomed to be disappointed again, more completely the second time than he was the first, for the fever was still higher and more clearly inflammatory in its character, while the suffering organ it was impossible to point out. The puzzle seemed to be impenetrable, and very naturally he became alarmed for the safety of his child and wanted counsel.

Upon my arrival the obscurity of the case was still the same. But upon one point we were agreed : The fever was clearly inflammatory. We did not then have the clinical thermometer, but the temperature could not have been estimated at less than 105°; pulse 120, full and bounding; face flushed and eyes brilliant.

I, therefore, proceeded to make a careful physical examination of every part of the body, *de fond en comble*, but with only negative results. No sign of local disease could be anywhere detected. But upon thinking over the different points of the case, I got back at length to the burn, and there I struck a trail that led us out of the woods. I thought of the similarity between a burn and an erysipelas, and then I remembered that I had seen the latter, when about cured, take a new departure from some points in its old track, and run over the whole of the inflamed surface again. Might it not do the same thing upon a burnt surface? And upon lifting a piece of the dry and loosened cuticle in this case, would not an erysipelas be found lurking beneath it?

This settled it. I announced the result of my thinking to Dr. Webb very much to his surprise and gratification, and we proceeded at once to uncover an erysipelas, exactly co-extensive with the surface blistered by the burn.

*Case* 2.—I was called in haste, August 12th, 1866, to see Mr. Z. T. H., æt. 45, a carpenter, tall and rather spare, but in good health usually, and found him lying upon the outside of his bed, with his clothes on, cold and pulseless, and in a state of profound insensibility.

His wife said : "I went out for a few minutes at about half past ten, leaving no one about the house but my husband, who was on the roof laying shingles. The roof looks to the South, and the sun was shining on it very hot; and when I returned in about half an hour, I found him here as you see him. He ate breakfast as usual and had not complained of feeling unwell."

In the alarm that naturally followed this discovery, several doctors had been called indiscriminately. Dr. K., an "eclectic," who arrived first, had called it a case of sunstroke and had, at once, began to apply hot fomentations to the extremities and to administer homœopathic dilutions. But in sunstroke, and also in apoplexy, in the former especially, the temperature is above the natural. We did not then have the clinical thermometer, but by taking his breath upon my face I was reasonably sure this man's heat was leaving him, and upon the whole, I was of the opinion that it was a case of pernicious fever of the algid type; and in this diagnosis Dr. Ashley, the family physician, who presently arrived, coincided, and we agreed also that the case was a critical one. But upon a vigorous resort to dry heat and mustard, quinine and capsicum, we had the pleasure, after a time, of seeing him slowly recover his bodily temperature and his consciousness.

But the rub came in the next morning. Upon calling to see our patient I found him still in bed, but he exclaimed that he had got about over his yesterday's sickness, and thought that in a little time he would feel strong enough to go to work again; and then with a sly twinkle in his eye he added:

"I suppose if I had been here myself I could have told you what the matter was yesterday."

"What do you say?" I said in some surprise, "were not we any of us smart enough to make out your case, after all?"

"I should say not," he replied, "but I will tell you what I know about it, and then you can judge. When I was on the roof yesterday, shingling, I was feeling as well as ever. The sun was shining on me, and it was pretty warm up there, but I was not aware that the heat was overcoming me in the least. Then, of a sudden, a big hornet came at me and run his stinger into my naked arm there; well! to judge by the hurt of it, I should say about a foot and a half, and it was not seemingly two minutes before a deathly sickness came over me which I cannot describe. I felt that I was done for sure enough. It appears that I got down off the roof and found my way into the house and on to the bed here, but how it was done, I have now no recollection."

Whose diagnosis was the correct one in this case, the patient's own or his medical attendants? Is the sting of an insect capable of producing, in some constitutions, the effects which we witnessed? Or were we correct in calling it a congestive chill, and the insect poisoning only an accidental complication? It seems the literature of such cases is meager. Can we not hear from some observer through the columns of the SOUTHERN MEDICAL RECORD?

*Case* 3.—In 1879, when the Ypsilanti and Hillsdale R.R. was building, I was called to see an Irishman who had just been dug out from under a bank of falling eartn. He was covered completely out of sight; but fortunately his cap was pushed down over his face in such a way as to retain around his mouth a few cubic inches of air, with which he was able to breath a little, and, what was more to the purpose, make an audible sound, for this smothered cry showed his comrades where his head was and saved his life.

I found him at his boarding-house, lying upon his back, not in much pain, not presenting the evidences of a profound shock and not under the impression that he was badly hurt, but complaining of great weakness in his lower extremities.

The men said they raised him to his feet when they got him out of the dirt, but his legs would not hold him up, and they thought the right limb seemed a little the weaker of the two. And the problem for me to solve was, what was the lesion upon which this weakness depended?

Was there a broken back? There was weakness enough perhaps to answer this question in the affirmative, but it was peculiar, it was not muscular. There was no paralysis of sensation, of motion or of organic life, and the bows of the legs were not broken; the hip joints were in place, and a careful manipulation of the pelvic bones failed to disclose any sign of weakness in them. And I was greatly at a loss for a rational opinion.

But the workmen said that when they shoveled him out, they found him lying upon his side, and just over his hips there was an unbroken lump of clay so heavy that it required the united strength of four men to roll it away, after the loose dirt around it had been removed. Here, possibly, was the clue of which I was in search. The immense pressure of this mass of earth, had been sufficient to fracture the pelvis at the symphysis pubis, but had been prevented from crushing the man fatally by the quantity of loose dirt which fell along with it. And I saw two ways in which this hypothesis could and probably would be verified.

In the first place, the fracture would be likely to take place exactly in front, that being the weakest point in the bone; it would necessarily be an oblique one, and the laceration and tumefaction of the soft parts beneath the bone and between it and the urethra would soon close that passage up. And this expectation was realized. Soon after the accident his urination was unobstructed; in ten hours more, it was necessary to use a catheter.

In the second place, the internal injury might be followed by a peritonitis. And this also was realized in like manner. The chill occurred in the early part of the first night, and the fever and other symptoms of a peritonal complication were unmistakable.

But the case did not prove to be a difficult one, except in the making of the diagnosis. The peritonitis did not spread, was probably limited by adhesions, and soon yielded to appropriate treatment. The use of a catheter twice a day was required for ten days, and in about four weeks, the time generally required for the consolidation of a broken bone, the patient was on his feet.

*Case* 4.—Mr. Ira W., æt. 50, commercial traveler, of active habits, and sound in body, as far as was known, called me on September 11, 1869. It was simply an attack of bilious remittent fever, for which I gave him a cathartic and followed its operation with quinine in two-grain doses every second hour, and the case went on very well. At 10 o'clock in the morning of the 12th, he had taken twenty-four grains of the sulphate, was free from fever and quite comfortable. And as his wife had not slept well the night before, he desired her to go to bed in another room, and leave him alone. And as he had no more medicine to take before morning, and seemed disposed to sleep himself, she did so.

In the morning, her first surprise was that he had not called for anything through the night. In some trepidation, therefore, she hurried to his bedside and was horrified to find him in a state of insensibility from which she could not arouse him.

What had happened to produce this sudden and unexpected change in my patient's condition? This was the question that confronted me when I entered the room a few minutes later, and without exception I think it was the most difficult diagnostic knot I was ever called upon to untie.

Was it the work of an opiate? My patient appeared to be narcotised, but upon this point the wife was very positive. No opiate had been prescribed, and she was sure there was no alcohol or other narcotic about the house which, in any possible way, he could have got hold of; and she did not believe he would have taken anything of the kind, even if he could. And it was not the coma of a fever—no fever was present. And it could not very well be an apoplexy, for the temperature was not above the normal, and the breathing was not stertorous. This disposed of four of the ordinary causes of a comatose condition, and there was but one more left to be considered—the uræmic. As to this, I had been his medical adviser for a number of years, and there was no previous history of any disorder of the kidneys whatever.

Upon turning this matter over, however, with my thinking cap on, it came to my mind that I had read somewhere that the sulphate of quinine had been found to produce comatose symptoms in a case of diabetes, by paralizing the function of a kidney already disordered and suspending the elimination of the urea. But where in this case was the diabetes, as a condition precedent? Could it be possible that this man had been passing diabetic urine and did not know it himself? His wife said he was not in the habit of leaving his bed for the purpose of micturition during the night, and she was not aware that he had been urinating more frequently or in greater quantity than would be natural. Could it possibly be a new accession of disease? There was no other clue at any rate, and I continued, therefore, to follow this one. The patient did not appear to have voided urine in a good many hours, and a supra-pubic dullness indicated a considerable accumulation of it. I drew it off, therefore, and tested it at my office. Its specific gravity was 1.037, and by Trammer's test, sugar was present in unmistakable quantity.

When this flow of diabetic urine began, we cannot know. The patient recovered his consciousness the next day. But the diabetes continued, and the patient died of phthisis in about three years.

I have recently had a case in consultation with Dr. H. B. Jenks, of Dentons, Michigan, where quinine in five-grain doses had been given to a diabetic patient, and no comatose symptoms had followed. But this man was in the very last stage of his disease. He died the next day.

